# The Influence of an AI-Driven Personalized Nutrition Program on the Human Gut Microbiome and Its Health Implications

**DOI:** 10.3390/nu17071260

**Published:** 2025-04-03

**Authors:** Konstantinos Rouskas, Mary Guela, Marianna Pantoura, Ioannis Pagkalos, Maria Hassapidou, Elena Lalama, Andreas F. H. Pfeiffer, Elise Decorte, Veronique Cornelissen, Saskia Wilson-Barnes, Kathryn Hart, Eugenio Mantovani, Sofia Balula Dias, Leontios Hadjileontiadis, Lazaros P. Gymnopoulos, Kosmas Dimitropoulos, Anagnostis Argiriou

**Affiliations:** 1 Institute of Applied Biosciences, Center for Research and Technology Hellas, 57001 Thessaloniki, Greece; rouskas@certh.gr (K.R.); mguela@certh.gr (M.G.); mpantoyra@certh.gr (M.P.); 2 Department of Food Science and Nutrition, University of the Aegean, Myrina, 81400 Lemnos, Greece; 3 Nutrition Information Systems Laboratory (NISLAB), Department of Nutritional Sciences and Dietetics, International Hellenic University, 57400 Thessaloniki, Greece; ipagkalos@ihu.gr (I.P.); mnhas@ihu.gr (M.H.); 4 Department of Endocrinology and Metabolic Diseases, Charité-Universitätsmedizin Berlin, 10117 Berlin, Germany; elena.lalama@charite.de (E.L.); andreas.pfeiffer@charite.de (A.F.H.P.); 5 Department of Rehabilitation Sciences, KU Leuven, 3001 Leuven, Belgium; elise.decorte@live.be (E.D.); veronique.cornelissen@kuleuven.be (V.C.); 6 School of Biosciences, Faculty of Health and Medical Sciences, University of Surrey, Guildford GU2 7XH, UK; s.wilson-barnes@surrey.ac.uk (S.W.-B.); k.hart@surrey.ac.uk (K.H.); 7 Research Group on Law, Science, Technology and Society, Faculty of Law & Criminology, Vrije Universiteit Brussel, 1050 Brussels, Belgium; eugenio.mantovani@vub.be; 8 Interdisciplinary Centre for the Study of Human Performance (CIPER), Faculdade de Motricidade Humana, Universidade de Lisboa, 1499-002 Lisbon, Portugal; sbalula@fmh.ulisboa.pt; 9 Department of Electrical and Computer Engineering, Aristotle University of Thessaloniki, 54124 Thessaloniki, Greece; leontios@auth.gr; 10 The Visual Computing Lab, Information Technologies Institute, Centre for Research and Technology Hellas, 57001 Thessaloniki, Greecedimitrop@iti.gr (K.D.)

**Keywords:** personalized nutrition, artificial intelligence, gut microbiome, human health

## Abstract

**Background/Objectives:** Personalized nutrition programs enhanced with artificial intelligence (AI)-based tools hold promising potential for the development of healthy and sustainable diets and for disease prevention. This study aimed to explore the impact of an AI-based personalized nutrition program on the gut microbiome of healthy individuals. **Methods:** An intervention using an AI-based mobile application for personalized nutrition was applied for six weeks. Fecal and blood samples from 29 healthy participants (females 52%, mean age 35 years) were collected at baseline and at six weeks. Gut microbiome through 16s ribosomal RNA (rRNA) amplicon sequencing, anthropometric and biochemical data were analyzed at both timepoints. Dietary assessment was performed using food frequency questionnaires. **Results:** A significant increase in richness (Chao1, 220.4 ± 58.5 vs. 241.5 ± 60.2, *p* = 0.024) and diversity (Faith’s phylogenetic diversity, 15.5 ± 3.3 vs. 17.3 ± 2.8, *p* = 0.0001) was found from pre- to post-intervention. Following the intervention, the relative abundance of genera associated with the reduction in cholesterol and heart disease risk (e.g., *Eubacterium coprostanoligenes group* and *Oscillobacter*) was significantly increased, while the abundance of inflammation-associated genera (e.g., *Eubacterium ruminantium group* and *Gastranaerophilales*) was decreased. Alterations in the abundance of several butyrate-producing genera were also found (e.g., increase in *Faecalibacterium*, decrease in *Bifidobacterium*). Further, a decrease in carbohydrate (272.2 ± 97.7 vs. 222.9 ± 80.5, *p* = 0.003) and protein (113.6 ± 38.8 vs. 98.6 ± 32.4, *p* = 0.011) intake, as well as a reduction in waist circumference (78.4 ± 12.1 vs. 77.2 ± 11.2, *p* = 0.023), was also seen. Changes in the abundance of *Oscillospiraceae_UCG_002* and *Lachnospiraceae_UCG_004* were positively associated with changes in olive oil intake (Rho = 0.57, *p* = 0.001) and levels of triglycerides (Rho = 0.56, *p* = 0.001). **Conclusions:** This study highlights the potential for an AI-based personalized nutrition program to influence the gut microbiome. More research is now needed to establish the use of gut microbiome-informed strategies for personalized nutrition.

## 1. Introduction

Diet and lifestyle changes can reduce risk for many chronic non-communicable diseases including obesity, diabetes, cardiovascular disease, and cancer [1]. However, the rates of diet-related diseases continue to grow and this may in part be due to the large variability in how people respond to foods [2,3], such that a single dietary strategy is not the most effective for everyone. Fortunately, personalized nutrition has now received great attention from the research community, moving away from the ‘one-diet-fits-all’ approach [4], and is emerging as one of the most revolutionary trends in the fields of health and food [5,6]. Moreover, tailored recommendations within personalized nutrition approaches may have beneficial effects on human health, leading to improvements in cardiometabolic health [7] and promoting well-being and healthy aging [8].

Simultaneously, there has been a big increase in the use of artificial intelligence (AI) technologies for nutrition in recent years with the potential to help food science and nutrition experts develop and promote sustainable, environmentally friendly [9,10], and ultimately personalized diets [11,12,13,14,15]. To date, there is some evidence for the utilization of AI in nutrition programs. For example, AI-driven chatbots have been shown to hold potential for designing diet plans for weight loss [16] and diabetes management [17]. Additionally, an evidence-based AI virtual dietitian was generated to address dietary questions for patients with cancer [18]. Furthermore, AI-assisted digital health applications such as an AI-supported continuous glucose monitoring mobile app have been proven to improve glycemic control in individuals with type 2 diabetes [19]. In the field of personalized nutrition, powerful AI-based digital health systems, including machine learning models, wearable sensors, and mobile applications, have been developed and are now being used for monitoring nutrition and generating dietary recommendations tailored to individual needs and characteristics [13,20,21,22,23,24,25]. However, their impact on distinct individual characteristics (e.g., lifestyle, biomedical, and gut microbiome data) as assessed through well-designed intervention studies remains poorly explored. Furthermore, current personalized nutrition programs underestimate the importance of biological factors associated with the large inter-individual and intra-individual variability observed in individual health responses to food [3]. Most of the personalized nutrition concepts have focused on genetic variants [26], but without adding evidence on how genetics influences dietary behavior, as shown in the Food4Me European study, the largest study of personalized nutrition to date [27,28]. Given the advances in the field of omics sciences, the exploration of other key biological factors such as the gut microbiome will offer unprecedented opportunities and open novel avenues in the field of personalized nutrition [29].

Among the biological characteristics relevant for personalized nutrition, the gut microbiome is an important factor that explains a large fraction of the physiological inter-individual variability [3,30,31,32]. The gut microbiome plays a key role in human health and disease [33] and shows a bidirectional relationship with diet, such that gut microbiome composition is shaped by diet [34,35,36,37], but also that the gut microbiome affects host metabolism and response to foods [38]. As a result, personalized nutrition interventions have been proposed to directly influence and modify the composition of the gut microbiome [31,39] and promote overall health. Personalized diets, for example, have been linked to improvements in cardiometabolic health [7] and cognition [40] by inducing beneficial shifts in gut microbiome composition. Furthermore, personalized nutritional interventions guided by microbiome profiles have also been gaining attention with promising outcomes [41]. Studies by Zeevi et al. [42] and Berry et al. [3], for example, developed personalized nutrition prediction models for postprandial glucose and triglycerides, respectively, based on gut microbiome features among others. In addition, two recent studies demonstrated the effectiveness of a microbiome-based AI-assisted personalized diet in the management of irritable bowel syndrome [43,44]. However, despite the high number of available nutritional mobile applications [45], very few studies such as the EU-funded Stance4Health project [46,47] explore the impact of personalized nutrition AI-based mobile apps on gut microbiome composition.

In the present study, as the primary objective, we evaluated the six-week effect of a dietary intervention program that uses an AI-driven mobile application for personalized nutrition on gut microbiota composition of healthy individuals. As a secondary objective, we determined changes in macronutrient and food group intake and anthropometric and biochemical traits and explored their associations with changes in the abundance of microbial genera.

## 2. Materials and Methods

### 2.1. Study Population and Design

**Figure 1:**
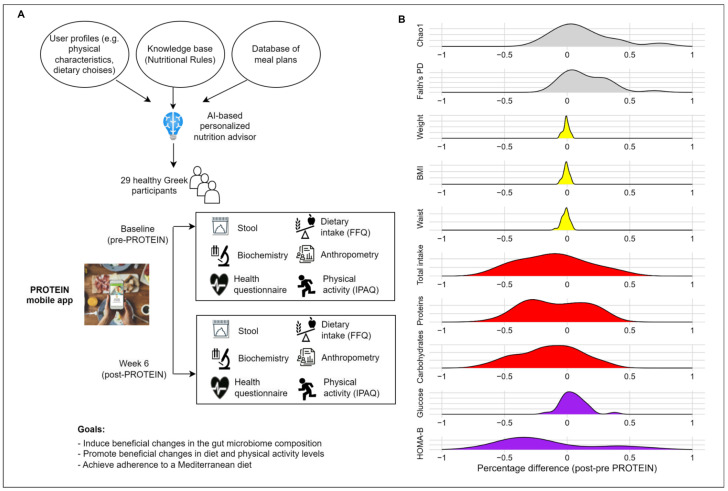
An overview of the PROTEIN study design and host parameters. (**A**) A schematic overview of the study design. Twenty-nine healthy participants used the personalized nutrition AI-based PROTEIN mobile app for six weeks to support beneficial changes to their diet and activity levels. A food frequency questionnaire (FFQ) and an International Physical Activity Questionnaire (IPAQ) were administered. Anthropometry measurements include height, weight, body fat percentage, muscular mass, basal metabolism, and hip and waist circumferences. (**B**) Density plots showing inter-individual variation in host variables in response to the PROTEIN dietary intervention. The X axis represents, for each variable and each individual, the ratio of the post–pre-PROTEIN values to the pre-PROTEIN values. Different colors represent metrics belonging to various studied traits i.e., grey: alpha diversity, yellow: anthropometry, red: diet and purple: biochemistry. AI, artificial intelligence; Faith’s PD, Faith’s phylogenetic diversity; BMI, body mass index; HOMA, homeostasis model for assessment of beta cell function.

This feasibility pilot study was conducted within the framework of an EU-funded project (i.e., PROTEIN: PeRsOnalized nutrition for hEalthy livINg, no 817732) that aims to promote a healthy lifestyle in the European population and offer personalized advice for nutrition and physical activity [21,22,24]. Twenty-nine apparently healthy individuals were recruited at the Center for Research and Technology (CERTH, Thessaloniki, Greece) for a six-week intervention. Participants were excluded if they had type 2 diabetes, cardiovascular disease, or any malignancy, followed a specific diet or food restriction, or had used antibiotics within the previous three months. All participants provided written informed consent to take part in this study, approved by the local ethics committee (ETH.COM-60, approved on 18 May 2021). An overview of the study design is shown in Figure 1A.

### 2.2. Intervention Using PROTEIN Mobile Application

Within the framework of the PROTEIN project, a digital smartphone health application was developed, aiming to engage people in a healthy, nutritionally sound, and active lifestyle. The PROTEIN mobile app generated daily and weekly meal plans through the use of a novel AI personalized nutritional advisor, validation of which is reported elsewhere [21,22,48]. The advisor takes into consideration the user profile (e.g., physical characteristics, dietary preferences, health conditions), population appropriate dietary recommendations, and a database of meal plans developed by a team of nutrition experts [49], as described in previous work [21,22,24]. In this prospective pilot study, a nutritionist implemented a six-week intervention with the participants using the mobile app (baseline; pre-PROTEIN and six-weeks follow-up; post-PROTEIN). At baseline, the nutritionist together with the participants set dietary and physical activity (PA) goals aiming to achieve adherence to the Mediterranean diet and a more active lifestyle considering each individual’s needs, with personalized nutrition and PA plans automatically generated by the AI advisor and delivered to users via the PROTEIN app. Participants were encouraged to follow the meal and activity plans provided every day via the smartphone application. At the six-week follow-up visit, the nutritionist evaluated their progress and repeated the baseline assessments. A study dietician was available for continuous support and feedback on participant progress throughout discussion and personal meetings.

### 2.3. Data Assessments

The following assessments were performed for all participants, before and after six weeks of the intervention with the PROTEIN mobile app.

#### 2.3.1. Dietary Intake

All individuals self-reported that they followed the diet of the general population before their participation in the study. Dietary data were collected via a validated semi-quantitative food frequency questionnaire (FFQ) comprising 79 questions [50] and designed to capture data on habitual diet over the previous month. We converted food consumption frequency into dietary data to determine the daily consumption in grams or milliliters (mL) as previously described in [50]. The reference serving size of each food item specified in the questionnaire was multiplied by the value related to each consumption frequency: never = 0; 1–3 times per month = 0.07; 1–2 times per week = 0.21; 3–6 times per week = 0.64; 1 time per day = 1; ≥2 times per day = 2. Through this process, we estimated the total daily energy and macronutrient (carbohydrate, protein, and fat) intake of each participant. Furthermore, we estimated separately the energy and macronutrient intake of each food group according to the FFQ with the use of the excel file of Athanasiadou et al. [51] and evaluated the adherence to the Mediterranean diet using the Mediterranean diet score (MDS) developed by Panagiotakos et al. [52]. Briefly, the MDS comprises 11 questions, ranges from 0 to 55 points, and scores the weekly consumption of non-refined cereals, potatoes, fruits, vegetables, legumes, fish, red meat and products, poultry, full fat dairy products, olive oil, and alcohol. Higher rankings in terms of the MDS correspond to higher adherence to the Mediterranean diet.

#### 2.3.2. Anthropometry and Body Composition Analysis

Anthropometric measurements were estimated by the use of the Tanita scale BC-545N, Tanita height measuring scale, and Tanita measuring tape. More specifically, height (m), weight (kg), body fat percentage, muscular mass (kg), basal metabolism (kcal/d), and hip and waist circumferences were evaluated. Body mass index was calculated as body weight divided by the square of height (kg/m^2^). Participants were defined as smokers and non-smokers (with past smokers and e-cigarette users also considered non-smokers).

#### 2.3.3. Physical Activity

To evaluate the PA levels of study participants, we used the validated International Physical Activity Questionnaire (IPAQ) short form, which comprises seven questions [53,54]. PA was classified as low, moderate, or high according to the IPAQ scoring system with the evaluation of metabolic equivalent intensity (MET) min/week, as previously described. Sedentary behavior was evaluated on the basis of the time spent sitting in the past seven days.

#### 2.3.4. Biochemical Blood Indices

Fasting blood samples were collected in the morning of the two study visits. Laboratory measurements including biochemistry (11 serum biomarkers) and complete blood count (CBC) testing (21 CBC traits) were performed at a private biomedical laboratory (‘Biodiagnosis’, Thessaloniki, Greece). The homeostasis model for assessment (HOMA) was calculated as a proxy for beta cell function (HOMA-B) and insulin resistance (IR). The equations used were as follows [55,56]:

HOMA-B = (360 * fasting insulin)/(fasting glucose − 63)

HOMA-IR = (fasting insulin × fasting glucose)/405.

The atheromatic index (total cholesterol/HDL cholesterol ratio) was also calculated to assess cardiovascular risk and risk of atherosclerosis [57].

### 2.4. Fecal Sample Collection, DNA Extraction, and 16S rRNA Amplicon Sequencing

Self-collection kits containing a preservation reagent (OMNIgene.GUT tube, DNA Genotek, Stittsville, ON, USA) were provided to study participants to collect fecal samples at home. The participants were instructed to collect samples within 24 h of their recruitment visit at baseline (pre-PROTEIN) and then at six weeks after the PROTEIN intervention (post-PROTEIN). The collected samples were delivered to the genomics facilities of the Institute of Applied Biosciences at CERTH for storage at −80 °C until processing. Fecal DNA extraction was performed using the ZymoBIOMICS DNA Miniprep Kit (Zymo Research, Irvine, CA, USA). The cell lysis and homogenization of fecal samples was conducted in Tissue Lyser II (Qiagen, Hilden, Germany) at 30 Hz for 5 min twice. All other steps were performed according to the manufacturer’s protocol. Quality control of extracted DNA was performed using Nanodrop and Qubit fluoremeter assays.

Gut microbiome composition and diversity were assessed by sequencing the V3–V4 hypervariable regions of the 16S rRNA gene (~460 bp) using the Illumina’s 16S Metagenomic Sequencing Library Preparation protocol. For the amplification of the V3–V4 region, gene-specific primers were selected based on Klindworth et al. (2013) [58] by adding Illumina overhang adapter nucleotide sequences at the 5′ end. Paired-end sequencing (2 × 300 cycles) of libraries was carried out on an Illumina MiSeq platform (Illumina Inc., San Diego, CA, USA). Amplicon sequences were demultiplexed based on index sequences, and fastq files were generated. Sequences were analyzed using the QIIME2 pipeline [59] and reads were denoised into amplicon sequence variants (ASVs) using DADA2 [60]. Taxonomy was assigned to ASVs against the SILVA 138 16S rRNA database [61] at a 99% threshold. Sequences classified as archaeal, chloroplastic, or mitochondrial were removed. The final ASV table (2354 ASVs in total) was imported into the phyloseq [62] package in R for downstream analyses.

### 2.5. Outcomes

The primary outcome of the present study was to assess pre- to post-PROTEIN intervention changes in the gut microbiome. Secondary outcomes encompassed changes in total energy intake, macronutrient consumption (carbohydrates, proteins, and fats), food group intake, anthropometric measurements, PA levels, biochemical markers, and any inter-relationships among these factors.

### 2.6. Statistical Analyses

Continuous traits were summarized by mean and standard deviation, while categorical variables were presented by count and percent prevalence. Linear mixed-effects models (nlme R package) including fixed effects for timepoint and a random effect for study participant were used to report changes in measured traits between timepoints, with post-PROTEIN as the reference timepoint. Prior to statistical analysis, we investigated the distribution for each trait, and 60 (out of 81 measured) non-normally distributed traits were log-transformed prior to regression analysis. Age and sex were used as fixed covariates in all association analyses. *p*-values <0.05 were considered statistically significant. The distribution of inter-individual changes in host variables in response to the PROTEIN intervention was shown through density plots, created using the geom_density function of ggplot R package [63]. For each variable and each individual, we calculated the relative percentage difference using the following equation:

Relative percentage difference = post − pre-PROTEIN/pre-PROTEIN

All statistical analyses were performed using R software (v 4.4.1).

The microbiome abundance matrix was rarefied to even depth (*n* = 12,397 reads per sample (minimum reads across the samples)) before the calculation of alpha and beta diversity indices. Three measures of alpha diversity were examined (i.e., Chao1, Pielou’s evenness and Faith’s Phylogenetic Diversity (PD)) in order to assess different aspects of alpha diversity, such as richness, evenness, and phylogenetic relatedness [64]. Alpha diversity measures were determined using R packages (microbiome, phyloseq, and picante), and differences across timepoints were tested using a Wilcoxon signed-rank test for paired data. Beta diversity was analyzed using both phylogenetic (weighted and unweighted Unifrac) and non-phylogenetic (Bray–Curtis dissimilarity) metrics. Permutational multivariate analysis of variance (PERMANOVA, 999 permutations) was conducted with the adonis2 function of the vegan (v2.6–4) R package (adonis2 function) to evaluate differences in the overall microbiome community structure across timepoints, using the subjects’ ID as strata for pairing. The results were plotted by principal coordinate analysis (PCoA) using the plot_ordination function of the phyloseq [62] R package.

Differential abundance of bacterial taxa between timepoints was identified with Maaslin2 package (v1.18.0) [65] accounting for sample pairing. ASV counts were filtered for a prevalence > 10% of total samples, rarefied to the minimum sequencing depth (n = 9484 reads) and transformed to relative abundance before running Maaslin2. The analysis was run with min.abundance = 0.01 and analysis.method = CPLM parameters, setting ‘timepoint as fixed effect and ‘subject ID’ as random effects and age and sex as covariates. The latter switched Maaslin2 analysis to linear mixed-effects model mode as it considers the longitudinal study design. The above prevalence and abundance filtering criteria resulted in a total of 221 ASVs as input for Maaslin2. The Benjamini–Hochberg procedure was used to correct *p* values, and corrected *p* values are reported as the false discovery rate (FDR). ASVs with a *p* < 0.05 and an FDR < 0.05 were considered differentially abundant.

To examine the functional capacity of participants’ gut microbiome, we applied the Phylogenetic Investigation of Communities by Reconstruction of Unobserved States (PICRUST2) pipeline [66] to predict MetaCyc pathways [67] from relative abundance data. Maaslin2 was used to identify differentially abundant pathways between timepoints. Compared to the differential abundance analysis of ASVs, Maaslin2 was run using the following different parameters: analysis.method = LM and transformation = LOG. Pathways with a *p* < 0.05 and an FDR < 0.05 were considered differentially abundant.

To identify associations among the collected metadata (anthropometric, dietary, biochemistry) and differentially abundant ASVs, Spearman’s rank correlations were calculated using the cor.test function in R. To reduce spurious correlations, rare features (prevalence < 50%) were filtered before correlating. Delta relative abundance matrices were constructed by subtracting, for each individual, the abundance of each differentially abundant ASV at baseline (pre-PROTEIN) from its abundance at the end of the intervention (post-PROTEIN). To reduce spurious correlations, only ASVs whose delta abundance differed from 0 in at least 10 individuals were retained for correlation analysis. R packages ggplot [63] and pheatmap [68] were used for plotting. Correlations with a *p*-value < 0.05 were considered statistically significant.

## 3. Results

### 3.1. Population Sample Description

Our population sample comprises 29 healthy individuals from Thessaloniki, Greece. In total, 52% of participants were female and had a mean ± SD age of 35 ± 8 years and body mass index (BMI) of 23.6 ± 3 kg/m^2^. Participants were not receiving any medication and most of them were non-smokers (82.8%). All participants were from families living above the poverty line [69]. Most of the participants were unmarried (65.5%) with tertiary education (86.2%) (Table S1). Regarding the dietary data, approximately 70% of individuals (20 out of 29) exceeded the daily energy intake recommended by the EFSA [70]). Furthermore, in regard to PA levels, 27.6%, 51.7%, and 20.7% of participants were assessed as having low, moderate, or high activity, respectively. The mean total IPAQ score had high variation as expressed by its standard deviation (1871.3 ± 2210.1 METs-minutes/week) and value range (146–11,724 MET-minutes/week), suggesting that it might not be informative for assessing inter-individual variation in PA levels. The average daily sitting time was 6.6 ± 3.6 h, with 44.8% of individuals classified as exhibiting sedentary behavior (≥8 h per weekday). All participants completed the six-week intervention.

### 3.2. Impact of Six-Week PROTEIN Intervention on Gut Microbiome

**Figure 2:**
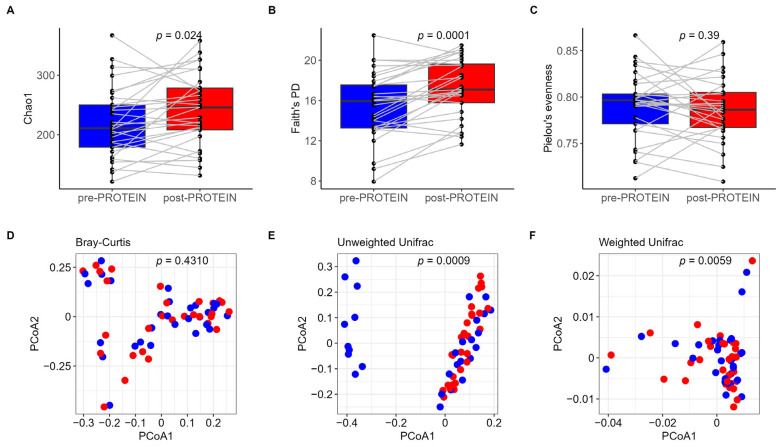
The impact of the PROTEIN dietary intervention on the alpha and beta diversity metrics. In the upper panel, boxplots of alpha diversity measures (**A**) Chao1, (**B**) Faith’s PD, and (**C**) Pielou’s evenness are shown. In the bottom panel, beta diversity is presented by PCoA, measured by non-phylogenetic (**D**) Bray–Curtis distance and phylogenetically aware, (**E**) unweighted and (**F**) weighted Unifrac distance metrics. Blue color corresponds to pre-PROTEIN and red color corresponds to post-PROTEIN metrics. *p*-Values for alpha and beta diversity metrics are from the Wilcoxon rank sum test and PERMANOVA test (999 permutations), respectively. PCoA, principal coordinate analysis; Faith’s PD, Faith’s phylogenetic diversity.

Sequencing of V3–V4 regions of 16S rRNA from 58 samples collected from 29 individuals yielded a total number of ~2.5 M reads, with an average depth per sample of 43,230 reads (ranging from 12,397 to 77,496 reads). In total, we identified 2354 ASVs that are assigned to 13 phyla, 19 classes, 44 orders, 82 families, and 231 genera. The gut microbiota of participants at baseline and the six-week follow-up timepoint were dominated by Firmicutes and Bacteroidota (Figure S1A), as is commonly observed in Western populations [71]. The most common genera at both timepoints were *Bacteroides*, *Faecalibacterium,* and *Prevotella* (Figure S1B). To investigate the impact of the PROTEIN intervention on the gut microbiome, alpha diversity metrics were calculated. We found that study participants at the post-PROTEIN timepoint had higher gut microbiota diversity and richness compared to baseline, as indicated by significant differences in Chao1 (*p* = 0.024, Figure 2A) and Faith’s PD (*p* = 0.0001, Figure 2B), respectively. No difference was found for Pielou’s evenness (*p* = 0.39, Figure 2C).

Non-phylogenetic and phylogenetically aware indices were used to assess the impact of the PROTEIN dietary intervention on the whole microbial composition. No significant difference in beta diversity, as measured by the Bray–Curtis dissimilarity metric, was observed (*p* = 0.431, Figure 2D). However, when phylogeny was taken into account, we found a significant difference in the unweighted Unifrac distance (*p* = 0.0009, Figure 2E), potentially attributable to low-abundance microbial ASVs contributing to the microbial community structure at the two timepoints. Because unweighted Unifrac distances consider only the presence and absence of microbial features, some pre-PROTEIN samples (10 out of 29) appeared to be less similar in community structure to the rest of the samples and formed a distinct cluster. When the abundance of ASVs was taken into account by using the weighted Unifrac distances, the majority of samples overlapped on the PCoA plot; however, the difference between timepoints remained significant (*p* = 0.0059, Figure 2F).

**Figure 3:**
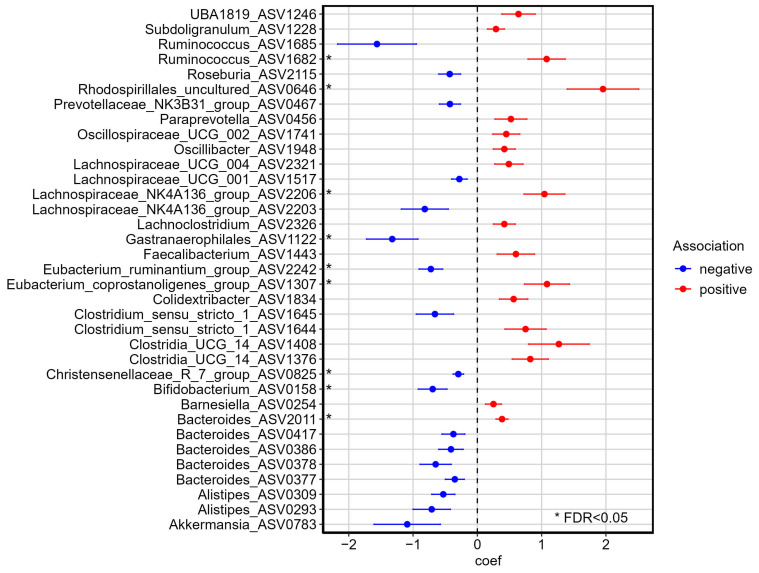
The associations of ASVs with the “timepoint” parameter of the MaAsLin2 multivariate regression model. The top 35 ASVs according to the significance of association are shown (*p* < 0.05). Associations with an FDR < 0.05 are indicated with an asterisk. Positive associations represent ASVs with increased abundance during the dietary intervention, while negative associations denote ASVs with decreased abundance. Error bars indicate standard deviation.

Having established pre- vs. post-PROTEIN differences in the level of the overall microbial community, we next sought to identify specific taxa that were affected by the PROTEIN intervention. Through differential abundance analysis, we identified nine bacterial ASVs that changed their relative abundance (FDR < 0.05, Maaslin2, Figure 3, Table S2). Five ASVs assigned to the *Bacteroides* genus were among the top thirty-five ASVs ranked by significance, of which four showed a tendency to decrease in abundance over the course of the dietary intervention. Among the 17 ASVs in Figure 3 that showed a trend to be downregulated by the intervention (*p* < 0.05), the largest coefficient belongs to an ASV assigned to *Ruminococcus*, but without reaching formal statistical significance (coef = −1.56, q = 0.14). The next two largest coefficients after *Ruminococcus* were observed for ASVs belonging to *Gastranaerophilales* (coef = −1.32, q = 0.03) and *Akkermansia* (coef = 1.09, q = 0.24) genera. By contrast, eighteen ASVs were upregulated by the intervention, of which fourteen belong to the Firmicutes phylum, which contains several butyrate-producing genera [72], including *Faecalibacterium* and *Lachnospiraceae_NK4A136_group*. The top responsive (upregulated) ASVs belonged to the top nine listed (FDR < 0.05), starting from *Rhodospirillales* (coef = 1.96, q = 0.02), followed by *Eubacterium coprostanoligenes group* (coef = 1.08, q = 0.05) and *Ruminococcus* (coef = 1.08, q = 0.01) genera.

To infer the functional potential of the observed taxonomic alterations, metagenomes were reconstructed using the PICRUSt2 algorithm. Of the 335 MetaCyc pathways predicted (Table S3), 12 pathways showed nominal significance (*p* < 0.05), mostly including microbial metabolic processes and purine degradation. However, none survived FDR < 0.05 correction.

### 3.3. Secondary Outcomes

**Figure 4:**
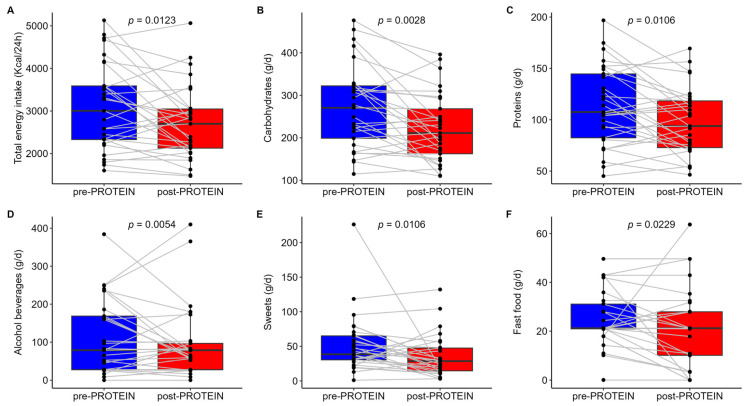
Boxplots of dietary factors significantly decreased after PROTEIN intervention. In the upper panel, daily intakes of (**A**) total energy, (**B**) carbohydrates and (**C**) proteins, at both timepoints, are shown. In the bottom panel, dairy intakes of food groups, (**D**) alcohol and beverages, (**E**) sweets and (**F**) fast food, at both timepoints, are shown. *p*-values were obtained from linear mixed models with participants (*n* = 29) as random effect, timepoint as fixed effect, and age and sex as covariates.

Mean values for all measured traits at both timepoints are reported in Table S4. Six weeks of intervention using the PROTEIN mobile app resulted in a significant decrease in carbohydrate (−18.1%), protein (−13.2%), and total energy (12.7%) intake (Figure 4A–C). The mean ± SD changes over the whole period of the intervention for carbohydrate, protein, and total energy intake were −49.3 ± 78.8 g, −15 ± 29.4 g, and −395.5 ± 824 Kcal, respectively. Substantial inter-individual variation was found in the PROTEIN-induced changes in macronutrient intake (Figure 1B). Information about the mean difference between timepoints and Δ% can be found in Table S4. Fat intake showed a similar trend (−12.5 ± 29.4 g) but was not statistically significant. When focused on specific food groups, we found a mean decrease of 39% in the intake of alcohol and beverages (mean ± SD change of −163.2 ± 257.7 g), a 33% decrease in sweets (mean ± SD change of −17.1 ± 45 g), and a 14% decrease in fast food (mean ± SD change of −3.6 ± 14.4 g) (Figure 4D–F). Salty snacks and dairy products showed a similar trend, without reaching statistical significance. On the contrary, we report a 62% increase in egg intake (mean ± SD change of 6 ± 14 g). Adherence to the Mediterranean diet did not change between timepoints, as shown by comparable values of the Mediterranean diet score (pre- vs. post-PROTEIN, 32.8 ± 3.7 vs. 33 ± 4.4, *p* = 0.812).

With regard to anthropometric measurements (Table S4), a small but significant reduction (−1.5%) in the mean waist circumference was found. More than half of participants (58.6%, 17 of 29 participants) showed a reduction in waist circumference, with a mean ± SD change from baseline of −1.2 ± 2.9 cm. Weight, BMI, and muscular mass showed non-significant decreases from baseline. PA levels, as assessed by the total IPAQ score, did not change at the end of the intervention. However, total percentages of individuals with low activity decreased from 27.6% to 13.8%, while percentages of individuals with moderate or high activity increased from 72.4% to 86.2%. At the individual level, five out of eight individuals with low activity at baseline shifted to the moderate activity group. Among biochemistry measurements (Table S4), we report an increase in blood glucose (+4.7%) and transaminases AST (+18.8%) and ALT (+17.9%) and a decrease (−26.1%) in HOMA-B. We also report statistically significant changes in 3 out of 21 CBC measured traits, with platelets displaying an increase (+5%) and MPV and PDW showing a decrease (−3.9% and −5.5%, respectively) at the end of the intervention.

To explore whether changes (post–pre-PROTEIN) in dietary factors affected by the intervention are associated with changes (post–pre-PROTEIN) in anthropometric and biochemistry traits, we performed Spearman’s correlation analysis (Figure S2). We identified 19 significant correlations (*p* < 0.05), of which 14 include changes in sweet and protein intake. In particular, changes in sweet intake were positively correlated with changes in weight, BMI, body fat, hip circumference, and hemoglobin and were negatively correlated with WHR. Protein intake showed a positive correlation with glucose, CRP, monocytes (counts and %), eosinophils (%), and basophils (counts and %) and a negative correlation with MCH. A change in total energy intake was positively correlated with monocytes, while carbohydrate intake showed a positive correlation with monocytes and basophils. A change in fast food intake was positively correlated with a change in HOMA-IR, while intake of alcohol and beverages was negatively correlated with weekly sitting hours. No correlation survived FDR < 0.05 correction, and thus, these correlation results have to be considered with caution.

### 3.4. Associations Between Gut Microbiota and Dietary, Anthropometric, and Biochemistry Variables

**Figure 5:**
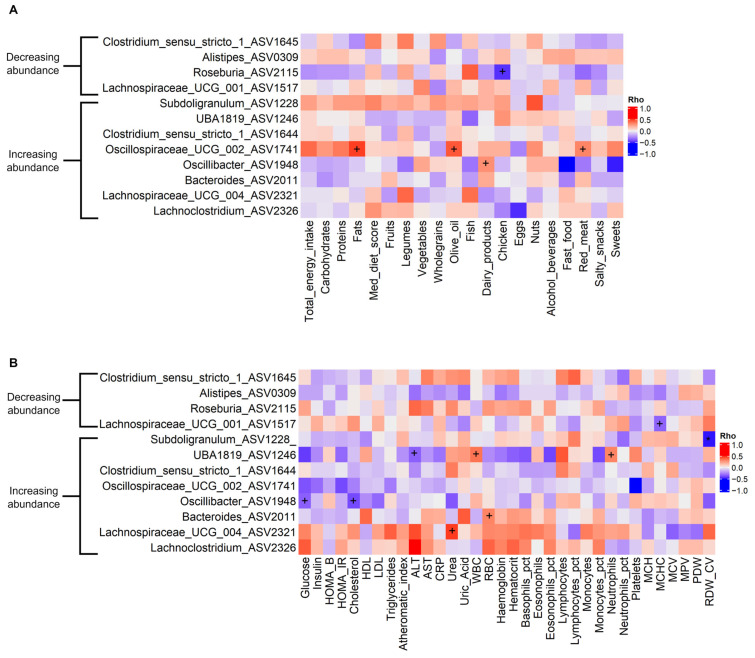
The correlation between differentially abundant genera and (**A**) dietary and (**B**) biochemistry traits at the post-PROTEIN timepoint. Significant correlations with Rho <|0.3| are not shown. Only prevalent (>50%) differentially abundant genera were tested in the correlation analysis. ^+^ *p* < 0.05 and * FDR < 0.05.

To identify specific interactions between microbial genera and dietary, anthropometric, and biochemistry variables, pairwise Spearman’s correlation was applied using data from the post-PROTEIN timepoint. In order to exclude false positive results, both the *p*-value and FDR are reported in Figure 5 and Table S5. Several correlations between genera and host variables were found with a *p*-value < 0.05. With regard to dietary variables, the strongest association was the positive correlation of fat intake with the abundance of *Oscillospiraceae_UCG_002* (Rho = 0.50, *p* = 0.031, q = 0.288). With regard to biochemistry variables, the strongest association was the negative correlation of RDW_CV with *Subdoligranulum*, which passed the FDR < 0.05 threshold (Rho = −0.50, *p* = 0.001, q = 0.033). The strongest positive correlation was the association of urea with *Lachnospiraceae_UCG_004* (Rho = 0.47, *p* = 0.022, q = 0.719). Of note, we found a negative correlation of cholesterol levels with *Oscillibacter* (Rho = −0.4, *p* = 0.048, q = 0.467), a genus previously reported as a cholesterol reducer [73]. No statistically significant correlations were found between anthropometric variables and microbial abundances.

**Figure 6:**
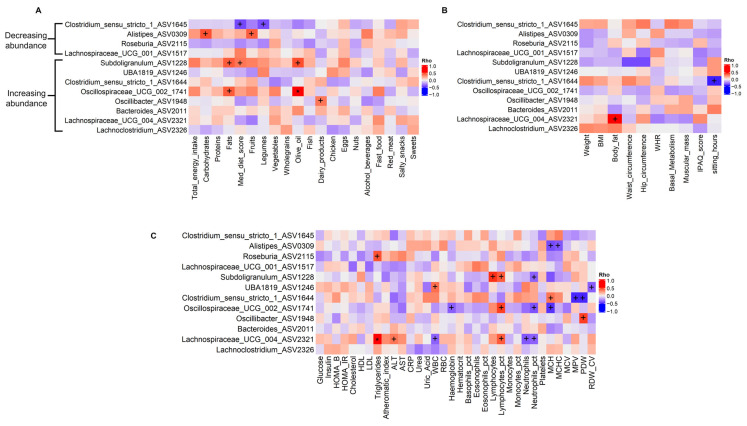
The correlation between changes (post–pre-PROTEIN) in differentially abundant genera and (**A**) dietary, (**B**) anthropometric, and (**C**) biochemistry traits. Only differentially abundant genera with a prevalence > 50% were tested in the correlation analysis. ^+^ *p* < 0.05 and * FDR < 0.05.

Finally, to control for individual variability in baseline host parameters, we performed Spearman’s correlation analysis between changes (post–pre-PROTEIN) in the relative abundance of differentially abundant genera and dietary, anthropometric, and biochemistry indices (Figure 6). With regard to dietary parameters, changes in olive oil intake were positively associated with changes in the abundance of *Oscillospiraceae_UCG_002* (Rho = 0.57, *p* = 0.001, q = 0.022). Changes in olive oil intake were also positively associated with changes in *Subdoligranulum*, albeit to a lesser degree (Rho = 0.48, *p* = 0.008, q = 0.131). Changes in carbohydrate, fat, and fruit intake also affected other genera, including *Oscillospiraceae_UCG_002, Subdoligranulum,* and *Alistipes*, without reaching statistical significance (q < 0.2). Among anthropometric measurements, changes in the abundance of *Lachnospiraceae_UCG_004* were positively associated with changes in body fat; however, this association did not reach statistical significance (Rho = 0.47, *p* = 0.010 q = 0.098). Among biochemistry variables, changes in triglycerides were positively correlated with changes in the abundance of *Lachnospiraceae_UCG_004* (Rho = 0.56, *p* = 0.001, q = 0.048). For CBC traits, the most significant associations observed were the negative correlation of *Clostridium_sensu_stricto_1* with MPV (Rho = −0.51, *p* = 0.005, q = 0.084) and PDW (Rho = −0.51, *p* = 0.005, q = 0.084). All correlation results, including Spearman’s Rho values and *p* and FDR-adjusted q-values, are reported in Table S6.

## 4. Discussion

This study aimed to evaluate the impact of an AI-based mobile application for personalized nutrition on the gut microbiome, as well as on the dietary, anthropometric, and biochemical characteristics of healthy individuals. Our findings contribute to a comprehensive understanding of the potential benefits of AI-assisted personalized dietary interventions for overall health.

### 4.1. PROTEIN Intervention and Overall Gut Microbiome Community Structure

After six weeks of dietary intervention designed to enhance adherence to the Mediterranean diet through personalized meal and activity plans, we observed a remarkable shift in the overall microbiota structure, as indicated by changes in alpha and beta diversity indices. Specifically, we found an increase in richness (Chao1) and phylogenetic diversity (Faith’s PD), as well as significant differences in beta diversity (measured by Unifrac distance metrics), highlighting the beneficial effects of the PROTEIN Mediterranean diet intervention on achieving a more diverse and balanced microbial environment. Our results are consistent with previous results from Bianchetti and colleagues’ four-week personalized nutrition trial in healthy Italian volunteers [74] and Bermingham and colleagues’ 18-week personalized dietary program in middle-aged healthy US individuals [7]. Similar improvements in the whole microbiome composition and diversity were also shown in studies exploring the impact of microbiome-based AI-assisted personalized diet on individuals with irritable bowel syndrome [43]. Microbial diversity has been extensively linked to several improvements in human health, with dietary interventions and personalized nutrition programs being considered significant contributors [41,75,76,77].

### 4.2. PROTEIN Intervention and Impact on Abundance of Genera of Interest

We identified several bacterial genera that exhibited significant changes in abundance following the PROTEIN dietary intervention. Among the top upregulated signals (Maaslin2, q < 0.05), we found an increase in the abundance of an ASV assigned to the *Eubacterium coprostanoligenes group* genus. This genus has recently received considerable attention due to its cholesterol-reducing properties and its potential influence on cardiovascular health [73]. Bacteria of this genus convert cholesterol into coprostanol, a less absorbable form, which may contribute to lower cholesterol levels in the body. Li and colleagues, through the Framingham Heart Study involving more than 1400 individuals [73], linked higher abundance of *Eubacterium coprostanoligenes group* and *Oscillibacter* to reduced cholesterol levels, as well as to potential benefits for lipid homeostasis and cardiovascular health. Notably, an increase in the abundance of an *Oscillibacter* ASV was observed following the PROTEIN dietary intervention (coef = 0.42, *p* = 0.013, q = 0.16), accompanied by a negative correlation with cholesterol levels at the post-PROTEIN timepoint. Personalized nutrition programs have recently been proven to be more effective in lowering cholesterol levels than general dietary advice [7]. Given the potential synergistic effects of *Eubacterium coprostanoligenes group* and *Oscillibacter* genera on cholesterol levels [73], it is suggested that personalized dietary interventions aiming to manipulate the gut microbiome could shed light on how different microbes interact to affect human health and also help individuals decrease their cholesterol levels and lower their cardiovascular risk.

Among the top downregulated signals (Maaslin2, q < 0.05), we revealed *Eubacterium ruminantium group* and *Gastranaerophilales* genera, which have been linked to various human diseases. *Eubacterium ruminantium* is an opportunistic pathogen that has been associated with an increased risk of multiple myeloma development [78] and respiratory diseases, potentially through the activation of an inflammatory response [79]. On the other hand, the bacteria *Gastranaerophilales* have been found to promote the production of plasma lipopolysaccharides (LPSs), which invade the circulation and cause inflammatory responses and cardiovascular diseases [80,81]. Further, the *Gastranaerophilales* genus showed an association with abdominal obesity represented by waist circumference [82]. Through personalized dietary interventions, we might achieve significant reductions in the abundance of pathogenic microbes and lead to improvements in overall human health; however, this requires further mechanistic investigation.

Short-chain fatty acids (SCFAs), including acetate, propionate, and butyrate, are gut microbial metabolites with beneficial health effects, with their levels influenced by environmental factors such as diet [83]. High intake of dietary fibers can lead to increased synthesis of gut-derived SCFAs [84], while a high-fat or a Western-type diet can reduce SCFA concentrations and alter gut microbiota composition in diet-related diseases such as obesity and metabolic syndrome [85]. Furthermore, adherence to the Mediterranean diet has been shown to favorably modulate levels of gut-derived SCFAs [86,87]. In our study, we also observed significant alterations in the abundance of several butyrate-producing genera. For example, *Faecalibacterium* and *Subdoligranulum* were increased post-PROTEIN, while *Bifidobacterium* and *Christensenellaceae R-7 group* were decreased. The *Faecalibacterium* genus and particularly the species *Faecalibacterium prausnitzii* represents one of the most abundant butyrate producers in adults, with lower levels associated with inflammatory conditions such as inflammatory bowel disease [88]. *Subdoligranulum* has been positively correlated with healthy metabolic status and could represent a promising probiotic candidate [89]. A reduction in *Bifidobacterium* is not surprising considering that these bacteria thrive on indigestive polysaccharides and that we found a decrease in carbohydrate intake post-PROTEIN intervention. A decrease in *Bifidobacterium* was also observed after a low-energy diet [90]. Furthermore, the decrease in *Christensenellaceae R-7 group* was likely promoted by the reduction in protein intake that occurred after the dietary intervention, as this bacterial group specializes in protein fermentation and may produce butyrate [91]. Members of the *Christensenellaceae* family have been linked to reduced visceral adiposity and a healthier metabolic profile [92]. The long-term impact of AI-based personalized nutrition programs on microbial capacity to produce butyrate requires clarification.

Recent evidence from the Personalised Responses to Dietary Composition Trial (PREDICT 1) supports the association of gut microbial signatures with multiple measures of dietary intake (food groups, nutrients, and dietary indices) and cardiometabolic health [34]. In our study, we found two interesting associations that passed the FDR < 0.05 cut-off and warrant further discussion. First, we found a positive correlation between changes (post–pre-PROTEIN) in *Oscillospiraceae_UCG_002* and the intake of olive oil, a basic component of the Mediterranean diet. Recent research indicated potential associations between olive oil intake and the abundance of microbes within the Ospillospiraceae family. For instance, a study focusing on the Mediterranean diet associated higher adherence with decreased abundance of *Oscillobacter* levels [93], while another study found a positive effect of olive oil intake on the abundance of *Faecalibacterium* [94]. Of note, *Oscillospiraceae_UCG_002* has been previously negatively correlated with HOMA-IR [95], a proxy of insulin resistance, which in turn has been found to decrease through a personalized nutrition program [96]. In addition, we found a positive association between changes (post–pre-PROTEIN) in *Lachnospiraceae_UCG_004* and levels of triglycerides. The role of this specific genus in triglyceride regulation has not been studied to date; however, several microbes belonging to the Lachnospiraceae family have been associated with altered lipid metabolism, and thus to obesity [97,98], or specific nutrients, such as saturated and total fats [99]. Given that the gut microbiota are key drivers of circulating lipid levels, including triglycerides [100,101], and affect host metabolism, our results contribute to this concept, although larger studies are needed to confirm our findings.

### 4.3. PROTEIN Intervention and Waist Circumference

The analysis of secondary outcomes revealed positive trends toward improved body composition, with a significant reduction in waist circumference. Other lifestyle intervention or personalized nutrition studies have found similar or greater changes in waist circumference; however, the duration of the interventions was larger, such as in the ZOE METHOD trial [7,102]. Conversely, a 12-week personalized nutrition trial in Chinese adults revealed no effects on waist circumference [103]. The small but statistically significant positive effect on waist circumference may reflect the impact of reducing multiple individualized postprandial responses and the greater satiating capacity reported by study participants. Measurements of waist circumference are vital in clinical practice, with even minor or moderate changes being associated with improved health outcomes [104].

### 4.4. Strengths, Limitations, and Future Perspectives

The present study has several strengths. First, this study evaluated the impact of an AI-based mobile application for personalized nutrition. Using digital health applications for tailoring dietary advice may advance human health and prevent chronic diseases. Second, we investigated the effects of a personalized nutrition program on several host factors including gut microbiome composition and parameters such as dietary habits and anthropometric and clinical traits. Finally, the study population consisted of younger individuals on average (mean age of 35 years with a range of 23–53 years) compared to other personalized nutrition studies [7], allowing the research community to consider practices of prevention of chronic diseases.

The results presented in this study should nonetheless be interpreted with some limitations in mind. First, the small sample size and lack of a control group limit our findings to associations. The smaller sample size of our study compared to other personalized nutrition studies [7] does not allow us to determine if the observed microbial alterations were driven by dietary changes or by psychological benefits of participating in a research study. The absence of a control group prevents us from concluding that our findings were due to the personalized nutrition or the use of an AI-based approach or a combination of these. However, our study was a Mediterranean diet intervention and exploring how this would be expected to impact the gut microbiome is important, regardless of how personalized advice is delivered to participants. To this end, we were able to reveal a significant shift in beneficial microbes, as shown in a larger PN study [7]. Second, the duration of this pilot study was short. However, given that microbiota may respond to diet within 1–3 days [39], a six-week framework should have been sufficient to observe microbiota changes. Further, we showed significant changes in measured dietary and biochemical traits, even with the short duration of the study. Studies with longer durations are needed to determine the effectiveness and durability of such PN interventions. Third, our study profiled gut microbiota through 16S rRNA amplicon sequencing, thus not allowing us to explore functional changes in the gut microbiota. Furthermore, we did not evaluate the effects of the PROTEIN intervention in subjects with chronic diseases. However, within the framework of the PROTEIN project, pilot studies in people with obesity or type 2 diabetes have been conducted [105], and similar research is needed in order to assess the effectiveness of AI-based personalized nutrition programs on health outcomes including weight loss and glucose level regulation. Finally, the assessment of dietary intake was based on a food frequency questionnaire (FFQ), which has limitations in capturing the intricate relations between diet and the gut microbiome [106,107].

Future research should focus on the development of personalized nutrition programs based on the integration of multiple-omics data including the microbiome, as well as genetic, metabolomics, and other data [29]. Furthermore, our intervention could be beneficial to healthy individuals but also may have long-term effects in the management and prevention of chronic diseases that in part are associated with gut dysbiosis such as obesity, diabetes, and cardiovascular disease. For example, the observed small but significant reduction in waist circumference may have beneficial effects against obesity in the long-term; however, longer follow-up periods are needed to validate this assumption. Moreover, personalized nutrition approaches, given their potential implication for the development and promotion of sustainable and environmentally friendly diets [108,109,110], should also consider environmental sustainability in dietary guidance. The promotion of dietary choices that are characterized by fewer animal products and more plant-based foods while decreasing food waste [111] is important. Given the advances in the use of AI-based technologies in agri-food systems [9,10], scaling personalized nutrition to a population-wide level may reduce waste, optimize health, and promote sustainability, inducing significant benefits in the food sector.

## 5. Conclusions

In conclusion, we provide evidence for a beneficial effect of an AI-based personalized nutrition program aimed at increasing Mediterranean diet adherence on the gut microbiome composition of healthy individuals. The importance of our findings can be shown by their potential practical implications at different levels. At the population level, beneficial changes in microbial abundances through the use of an AI-driven dietary program may lead to improvements in the gut microbial ecosystem and reduce issues like bloating, constipation, or inflammatory bowel syndrome. Moreover, enhancement of the immune system and better tolerance to infections and autoimmune diseases may be achieved. Of note, performance of similar efforts in people suffering from chronic diseases such as obesity, diabetes, and cardiovascular disease would help us move toward better prevention of disease, reduced healthcare costs, and improved overall public health. Our results could also inform gut microbiome-guided dietary interventions and their inclusion in everyday clinical practice aiming to promote health and reduce disease risk. For the scientific community, the present study adds to current knowledge and helps in deepening the understanding of the links between diet, the gut microbiome, and health. Moreover, our study may help identify key microbial biomarkers that correlate with disease outcomes. Ultimately, such AI-driven programs may facilitate research into food-based therapies by identifying dietary interventions that support a healthy microbiome and advance the use of the food as a medicinal approach. Studies of longer follow-up periods and larger sample sizes integrating additional biological layers of information such as genetics, transcriptomics, and metabolomics would enable a more comprehensive and holistic evaluation of the impact of AI-based personalized nutrition approaches on health outcomes.

## Data Availability

The raw sequence files are available at the NCBI Sequence Read Archive (https://www.ncbi.nlm.nih.gov/sra/, assessed on 31 March 2025) under BioProject ID number PRJNA1227288.

## Supplementary Materials

The following supporting information can be downloaded at https://www.mdpi.com/article/10.3390/nu17071260/s1: Figure S1: Compositional plot of relative abundance of top ten (A) phyla and (B) genera at two timepoints. Figure S2: Correlation heatmap of changes (post–pre-PROTEIN) between dietary factors that altered post-intervention and anthropometric or biochemistry measurements. Table S1: Characteristics of study individuals. Table S2: Differential abundance analysis of ASVs between pre- and post-PROTEIN timepoints. Table S3: Differential abundance analysis of MetaCyc pathways between pre- and post-PROTEIN timepoints. Table S4: Changes in measured traits between pre- and post-PROTEIN timepoints. Table S5: Spearman’s correlations between prevalent (>50%) differentially abundant genera and measured variables at post-PROTEIN timepoint. Table S6: Spearman’s correlations between changes in prevalent (>50%) differentially abundant genera and measured variables.

## Abbreviations

The following abbreviations are used in this manuscript:

AI	artificial intelligence
PROTEIN	PeRsOnalized nutrition for hEalthy livINg
FFQ	food frequency questionnaire
IPAQ	International Physical Activity Questionnaire
BMI	body mass index
HOMA	homeostasis model assessment
MDS	Mediterranean diet score
ASV	amplicon sequence variant
PICRUST2	Phylogenetic Investigation of Communities by Reconstruction of Unobserved States
PCoA	principal coordinates analysis
FDR	false discovery rate
PA	physical activity
DA	differentially abundant
