# Supplementary figures and images for: The Influence of an AI-Driven Personalized Nutrition Program on the Human Gut Microbiome and Its Health Implications
Source: Nutrients. 2025 Apr 3;17(7):1260. doi: 10.3390/nu17071260 (PMC11990151; doi:10.3390/nu17071260)

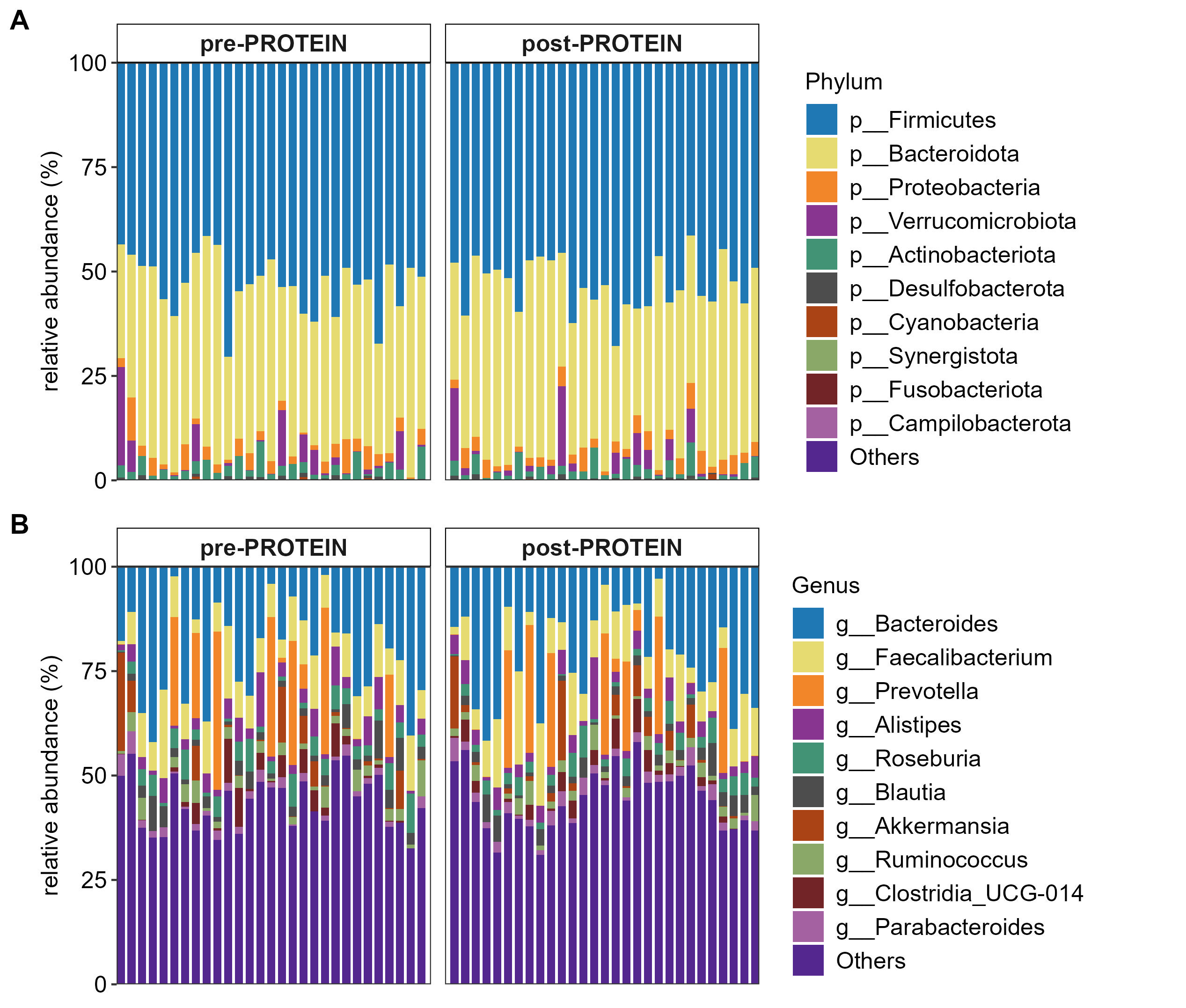

Supplement: Supplementary file 1 [file nutrients-17-01260-s001.zip › FigureS1.jpeg]

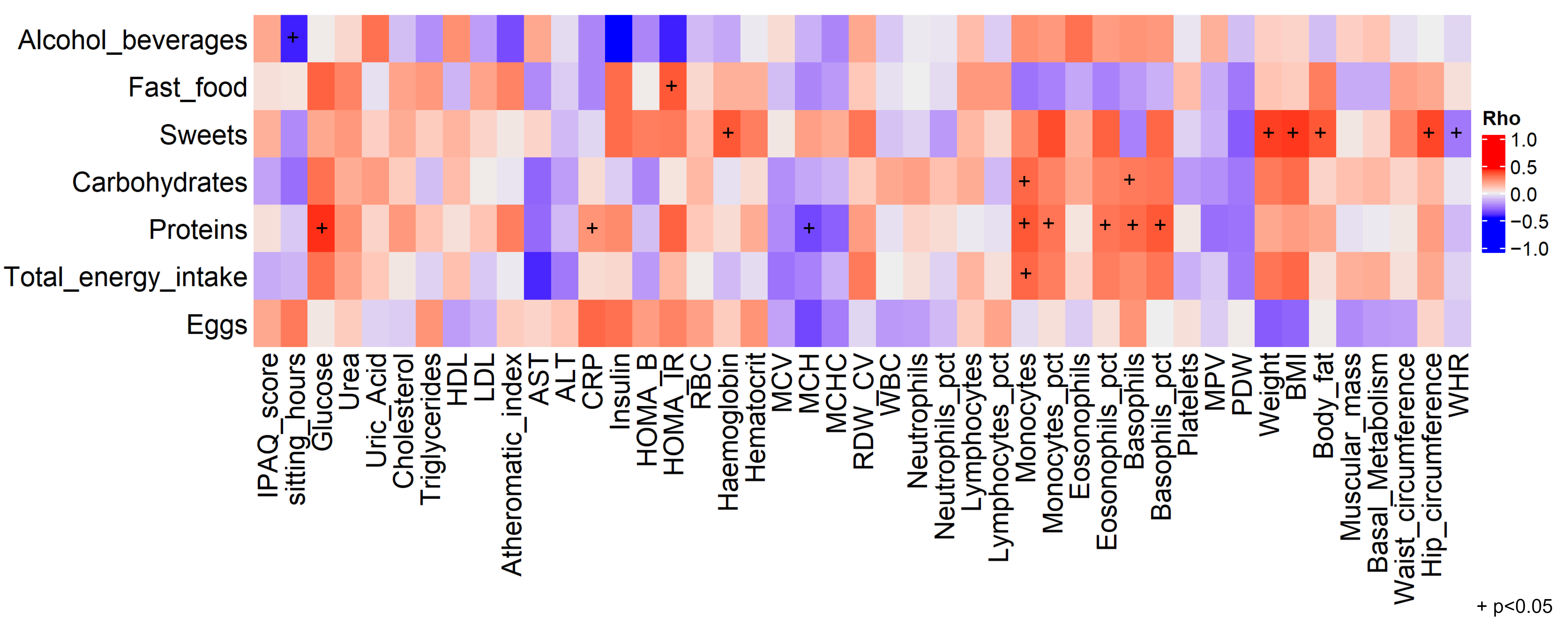

Supplement: Supplementary file 1 [file nutrients-17-01260-s001.zip › FigureS2_final.png]
